# Evaluation of chronic toxicity and carcinogenicity of ammonium 2,3,3,3-tetrafluoro-2-(heptafluoropropoxy)-propanoate in Sprague–Dawley rats

**DOI:** 10.1016/j.toxrep.2015.06.001

**Published:** 2015-06-30

**Authors:** J.M. Caverly Rae, Lisa Craig, Theodore W. Slone, Steven R. Frame, L.William Buxton, Gerald L. Kennedy

**Affiliations:** a E I. du Pont de Nemours and Company, Inc., Haskell Global Centers for Health & Environmental Sciences, Newark, DE 19714, USA; b MPI Research, Inc., Mattawan, MI 49071, USA; c E I. duPont de Nemours and Company, Inc., Chemicals and Fluoroproducts, Wilmington, DE 19805, USA; d Consultant to DuPont Company, Wilmington, DE 19805,USA

**Keywords:** Fluoropolymers, Chronic toxicity and carcinogenicity study, Sprague–Dawley rats, PPARα agonist

## Abstract

Ammonium 2,3,3,3-tetrafluoro-2-(heptafluoropropoxy)-propanoate, developed for use as a polymerization processing aid in the manufacture of fluoropolymers, was tested for its potential chronic toxicity and carcinogenicity in a 2-year oral dosing study in Sprague–Dawley rats. Male rats were given daily doses of either 0, 0.1, 1 or 50 mg/kg; females were given either 0, 1, 50 or 500 mg/kg. Body weights, food consumption and clinical signs were monitored daily; clinical pathology was conducted at designated intervals and animals were given a complete pathological evaluation after 12 months and 24 months of dosing. Normal survival was seen in all groups, no abnormal clinical signs were seen, and body weight gain was reduced only in female rats at 500 mg/kg. Both sexes at the high dose had mild decreases in red cell mass which were somewhat more pronounced in females. Clinical pathology indicative of liver injury was present in males that received 50 mg/kg and correlated with histomorphological liver changes that included both hypertrophic and degenerative/necrotic lesions. Similar histomorphological lesions were seen in the livers of females at 500 mg/kg. Previous shorter term toxicity studies have identified this chemical as a PPARα agonist and the finding of benign tumors of the liver, pancreas and/or testes in males at 50 mg/kg and females at 500 mg/kg is consistent with the rat response to peroxisome proliferators and is of questionable human relevance. Changes in the kidney, tongue, and stomach were observed only at the highest dose of 500 mg/kg in females. The no-observed-adverse-effect-level in this study lies between 1 and 50 mg/kg for males and between 50 and 500 mg/kg for females.

## 1 Introduction

Ammonium 2,3,3,3-tetrafluoro-2-(heptafluoropropoxy)-propanoate (CAS 62037-80-3) is a white/colorless solid with a sublimation point of 130–140 °C, a decomposition point of 150–160 °C, a density of approximately 1.7 g/cm^3^ at 20 °C, and a very low vapor pressure of approximately 0.01 Pa at 20 °C [10]. The chemical has been developed for use as a polymerization processing aid in the manufacture of fluoropolymers. The acute toxicity profile includes both an oral LD50 of 1750 and 3129 mg/kg in male and female rats, respectively, and 1030 mg/kg in mice (females). The dermal LD50 in rats is greater than 5000 mg/kg. By the inhalation route, the chemical has an acute LC50 of greater than 5200 mg/m^3^ in rats exposed for 4 h. As tested in rabbits, it is highly irritating to the eye but is not a skin irritant nor is it a sensitizer as tested by the mouse local lymph node assay. The material is not genotoxic based on a battery of tests including Ames and chromosome aberration in-vitro studies and mouse micronucleus, mouse bone marrow chromosomal analysis and rat unscheduled DNA synthesis in-vivo studies [10].

The compound is rapidly and completely absorbed following oral administration in both rats and mice, is not metabolized, and is eliminated almost exclusively in the urine. In the cynomolgus monkey given a single intravenous dose, the chemical was rapidly eliminated, no sex differences were seen, and a biphasic pattern of elimination was present. Based on oral studies in rats and mice, and intravenous studies in rats and primates, elimination of the test substance is most similar and more rapid in primates and rats, with mice having a comparatively longer clearance time. Clearance times in rats and primates are generally less than 12–24 h while those in mice are 60–140 h. Plasma elimination kinetics were similar following both single and multiple dose studies indicating a low propensity for accumulation in the body. Based on the pharmacokinetic curve following an intravenous dose of the compound, male rats have a slightly slower elimination rate than females, resulting in males having a higher plasma concentration than females after the same dose [11].

In repeated dose toxicity studies, the test material induces hepatic peroxisomal β-oxidation activity and produces microscopic changes in the livers of rodents consistent with the activity of peroxisome proliferators. Thus, the test material is a member of a class of compounds known as PPARα agonists, which include endogenous long chain fatty acids, as well as many industrial chemicals and pharmaceuticals. These compounds produce effects in the liver to which rodents have been shown to be more sensitive than other species, including humans. In a series of repeated oral dosing studies of 28–90 days duration in rats and mice, the sentinel effects of this chemical were consistent with those of a PPARα agonist [12], [13]. These included increased liver beta-oxidation activity, increased liver weights, microscopic hepatocellular hypertrophy, and clinical pathology changes of decreases in red cell parameters, changes in serum proteins, and decreases in serum lipids.

Specifically, in a 90-day repeated dose toxicity study in which male rats were given daily gavage doses of either 0.1, 10, or 100 mg/kg, findings included most of the clinical pathology and anatomic pathology effects noted above, as well as increased kidney weights [13]. Similar finding were seen in female rats given daily gavage doses of either 10, 100, or 1000 mg/kg. These included kidney weight increases at 10 mg/kg, PPARα-related clinical chemistry changes at 100 mg/kg, and red blood cell effects, increased liver weights, and hepatocellular hypertrophy at 1000 mg/kg. Findings similar to these were seen in an earlier 28-day dosing study with liver effects seen in males following dosing at 3 mg/kg and in females at 300 mg/kg [12]. These studies formed the basis for dose level selection for the chronic toxicity/carcinogenicity study reported here.

To characterize the chronic toxicity profile and to evaluate the potential carcinogenic effects of this chemical, a 2-year chronic toxicity and carcinogenicity study was conducted in rats. This paper covers the information derived from this experiment. The results of this study are reported herein and will be compared to those found in other chronic/carcinogenicity studies with similar fluorochemicals to place the findings in perspective.

## 2 Materials and methods

### 2.1 Chemicals

The ammonium salt of 2,3,3,3-tetrafluoro-2-(heptafluoropropoxy)-propionic acid (84%; CAS number 62037-80-3; C_6_H_4_F_11_NO_3_; molecular weight: 347) was provided by E.I. du Pont de Nemours and Company (Wilmington, DE). The test article was an 84% aqueous solution and doses were adjusted accordingly and are expressed in terms of 100% test chemical. Formulations of the test article were prepared weekly and stored at room temperature.

### 2.2 Animals

All rat treatments, toxicity assessments and data acquisitions were performed at MPI Research, Inc. (Mattawan, Michigan). The protocol was reviewed and approved by the Institutional Animal Care and Use Committee (IACUC), in compliance with the Animal Welfare Act (AWA). Male and female CD^®^ [Crl:CD(SD)] rats were received at approximately 5 weeks of age from Charles River Laboratories (Portage, Michigan). Rats were acclimated for 14 days prior to being randomly assigned to treatment groups using a standard, by weight, measured value randomization procedure. Rats assigned to study had body weights within ±20% of the mean body weight for each sex. The animals were pair-housed (same sex) in polyboxes with non-aromatic bedding in an environmentally-controlled room and were provided environmental enrichment. All animals were maintained in accordance with the Guide for the Care and Use of Laboratory Animals in the animal facilities at MPI Research, Inc. (American Association for the Accreditation of Laboratory Animal Care [AAALAC] accredited). Block Lab Diet^®^ (Certified Rodent Diet #5002, PMI Nutrition International, Inc., Shoreview, MN, USA) was provided ad libitum. Tap water was available ad libitum via an automatic watering system. Room temperature and humidity controls were maintained to the maximum extent possible between 64 and 79 °C and 30–70%, respectively. A 12 h light/dark photoperiod was maintained.

### 2.3 Treatment

Four treatment groups of 80 rats/sex were formed. Control group males and females received deionized water (vehicle control). Male rats in test groups received either 0.1, 1 or 50 mg ammonium 2,3,3,3-tetrafluoro-2-(heptafluoropropoxy)-propanoate/kg body weight/day. Female rats in test groups received either 1, 50 or 500 mg ammonium 2,3,3,3-tetrafluoro-2-(heptafluoropropoxy)-propanoate/kg body weight/day. Ten animals/sex/group were designated for the 12 month interim necropsy. The remaining surviving animals were designated for the terminal necropsy.

Dose selection was based on a previous subchronic (90-day) toxicity study [13] and a previous 28-day study [12]. The vehicle and test article were administered once daily for up to 104 weeks in males and up to 101 weeks in females via oral gavage at a dose volume of 10 mL/kg/dose. The control group received the vehicle in the same manner as the treated groups. Individual doses were based on the most recent body weights. All animals were observed for morbidity, mortality, injury, and availability of food and water twice daily. Beginning at week 53, a third daily cageside observation was added. A detailed clinical examination, including a physical exam and palpation of masses, was performed weekly. Ophthalmoscopic examinations were conducted on all animals pretest and on all surviving animals prior to the interim and terminal necropsies.

### 2.4 Body weight and food consumption

Body weights were recorded weekly starting day 1 (prior to dosing) until week 14, and then every two weeks thereafter. Body weight changes were calculated and reported weekly the first 3 months (weeks 1–13), the first year (weeks 1–52), and for the entire study (weeks 1–102 for males and weeks 1–100 for females). Food consumption was recorded pretest (week – 1), weekly during the first 13 weeks, and then every two weeks starting on week 14. Food consumption was measured for the cage and divided by the number of surviving animals. Food consumption and efficiency were calculated weekly the first 3 months (weeks 1–13), the first year (weeks 1–52), and for the entire study (weeks 1–102 for males and weeks 1–100 for females).

### 2.5 Clinical pathology

Clinical pathology evaluations were conducted on 10 animals/sex/group at 3, 6 and 12 months (hematology and clinical chemistry) and 10 animals/sex/group at 6 and 12 months (coagulation and urinalysis). The first ten animals in each group were selected for bleeding and these same animals were used for all time points. Blood samples were collected via the vena cava or cardiac puncture after carbon dioxide inhalation. The order of bleeding was by alternating 1 animal from each dose group then repeating to reduce handling and time biases. Blood samples for peripheral blood smears were collected at 12 and 18 months and prior to terminal necropsy. The animals designated for a full clinical pathology evaluation had access to drinking water but were fasted overnight prior to scheduled sample collection. The animals designated for only blood smears had access to drinking water and food prior to sample collection. Urine was collected after animals were housed in stainless steel metabolism cages for at least 12 h.

Hematology parameters evaluated included: leukocyte count (total and absolute differential), erythrocyte count, hemoglobin, hematocrit, mean corpuscular hemoglobin, mean corpuscular volume, mean corpuscular hemoglobin concentration (calculated), absolute reticulocytes, platelet count, and platelet morphology. Coagulation parameters evaluated included prothrombin time and activated partial thromboplastin time.

Clinical chemistry parameters evaluated included alanine aminotransferase, alkaline phosphatase, sorbitol dehydrogenase, total protein, albumin, globulin and A/G (albumin/globulin) ratio (calculated), urea nitrogen, creatinine, total cholesterol, triglycerides, total bilirubin (with direct bilirubin if total bilirubin exceeded 1 mg/dL), aspartate aminotransferase, total bile acids, glucose, calcium, phosphorus, electrolytes (sodium, potassium and chloride), and gamma glutamyl transferase.

Urinalysis parameters evaluated included volume, specific gravity, pH, color and appearance, protein, glucose, bilirubin, ketones, blood, urobilogen, and microscopy of centrifuged sediment.

### 2.6 Necropsy and histopathological evaluation

Postmortem study evaluations were performed on all animals euthanized in extremis, animals found dead, and on all surviving animals scheduled and sacrificed at the interim (12 months) and terminal (101 and 104 weeks – approximately 24 months) necropsies. The animals were euthanized by carbon dioxide inhalation followed by exsanguination via the abdominal vena cava. At necropsy, external surfaces and abdominal, thoracic, and cranial cavities were examined for abnormalities including masses. Tissues from all organ systems (digestive, urinary, respiratory, cardiovascular, hematologic, nervous, endocrine, musculoskeletal, and reproductive systems, as well as skin and gross observations) were removed, examined, and, where required by protocol, placed in fixative (neutral buffered formalin, except for the eye [including the retina and optic nerve] and testes, which were fixed using a modified Davidson’s fixative). Body weights and organ weights (brain, adrenals, heart, kidneys, liver, spleen, thyroid/parathyroid, and/or epididymides, testes, ovaries with oviducts, and uterus with cervix) were recorded for all surviving animals at the scheduled necropsies and appropriate organ weight ratios were calculated (relative to body and brain weights). Paired organs were weighed together. Microscopic examination of fixed hematoxylin and eosin-stained paraffin sections was performed on tissues by a board-certified veterinary pathologist. A four-step grading system was utilized to define gradable lesions for comparison between dose groups. For fatal and incidental neoplasms, the onset date was considered to be the fate date of the affected animal. For mortality independent neoplasms, the onset date was considered to be the first appearance of a related abnormality (e.g., abrasion, nodule, and/or swelling). A second board-certified pathologist performed a formal peer review of the histopathological findings and the results reported here represent the consensus.

### 2.7 Statistical analysis

For continuous data (body weight and weight gain, food consumption, hematology, coagulation, clinical chemistries, and organ weights), the statistical procedures were generally as follows: Levene’s test [20] was used to assess homogeneity of group variances for each specified endpoint and for all collection intervals. If Levene’s test was not significant (*p* > 0.01), a pooled estimate of the variance (mean square error) was computed from a one-way analysis of variance (ANOVA) and utilized by a Dunnett’s comparison [9] of each treatment group with the control group. If Levene’s test was significant (*p* < 0.01), comparisons with the control group were made using Welch’s *t*-test [28] with a Bonferroni correction.

Intercurrent mortality data were analyzed using the Kaplan–Meier product-limit method. An overall test comparing all groups was conducted using a log-rank test [2]. Tumor incidence data were analyzed using both survival adjusted and unadjusted tests. The Cochran–Armitage trend test [1] was calculated and Fisher’s exact test [29] was used to compare each treatment group with the control group. The survival adjusted test was conducted according to the prevalence/mortality methods described by Peto et al. [24].

All endpoints were analyzed using two-tailed tests with results of all pair-wise comparisons reported at the 0.05 and 0.01 significance levels.

## 3 Results

This experiment was conducted in accord with the Toxic Substance Control Act (TSCA) Good Laboratory Practice Standards. All system suitability tests, performance checks, and calibration standards met analysis acceptance criteria and the dose formulation samples, analyzed for homogeneity and concentration, met the criteria for acceptance based on accuracy and precision. On quality assurance check, no significant deviations from the protocol which could impact the quality or integrity of the study were found.

### 3.1 Survival

**Fig. 1:**
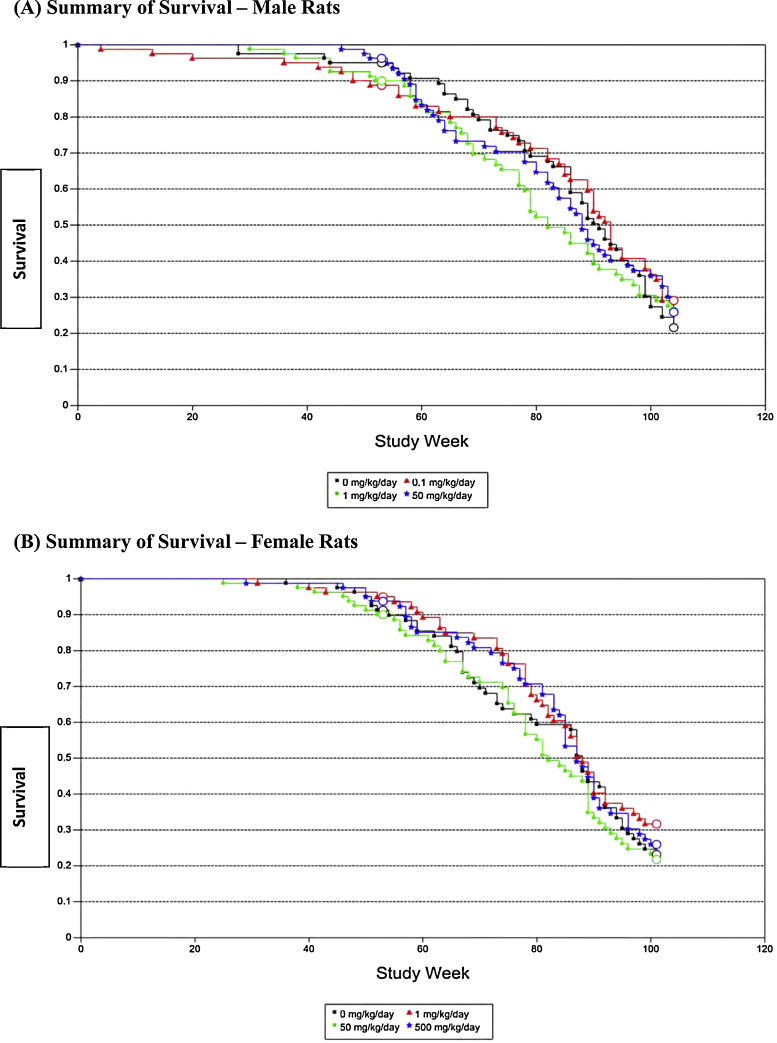
Kaplan–Meier survival curves for rats treated with ammonium 2,3,3,3-tetrafluoro-2-(heptafluoropropoxy)-propanoate for 104 weeks (male) or 101 weeks (female). (A) Survival curve of male rats treated with either 0, 0.1, 1 or 50 mg/kg. *p* > 0.05. (B) Survival curve of female rats treated with either 0, 1, 50 or 500 mg/kg, *p* > 0.05.

Mean survival data for male and female rats are illustrated in Fig. 1. The test substance had no effect on survival. Females were sacrificed during week 101, prior to scheduled termination at week 104, due to low survival in all female dose groups (especially control and 50 mg/kg groups). However, even though survival among all female groups was low, there were no statistically significant differences between groups as survival was essentially the same.

Seven of the 500 mg/kg early death females had test substance-associated papillary necrosis and inflammation of the kidney. All other causes of death/morbidity were considered incidental and common in rats of this strain and age.

### 3.2 In-life observations

There were no test substance-related clinical observations; all observations were transient or common in this species. In addition, there were no test substance-related increases in masses identified during physical examination nor were there any test substance-related findings at the interim or terminal ophthalmoscopic examinations.

The test substance had no effect on body weight, body weight gain, or food consumption and efficiency in males. These values were generally comparable to those of control animals throughout the study.

**Fig. 2:**
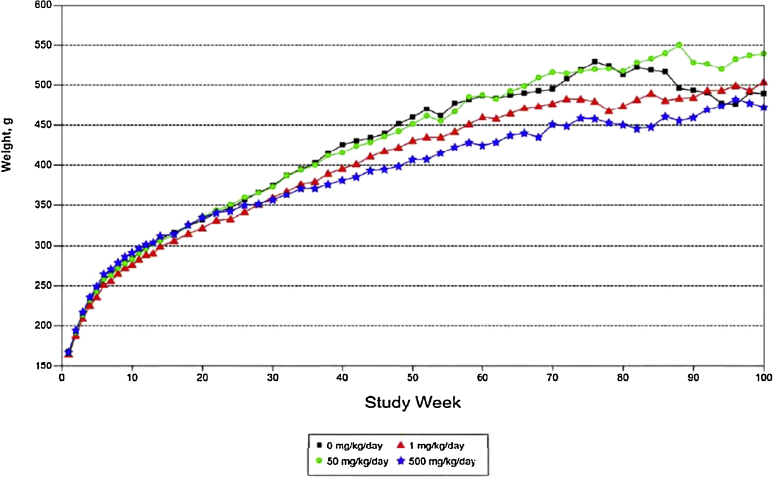
Mean body weights for female rats treated with either 0, 1, 50 or 500 mg/kg ammonium 2,3,3,3-tetrafluoro-2-(heptafluoropropoxy)-propanoate for 101 weeks.

Exposure of female rats to 500 mg/kg produced reductions in body weight, body weight gain, and food efficiency. Mean body weight for this group was 13% below that for control animals at week 52 (statistically significant), but was comparable to controls at study termination (Fig. 2). Consequently, mean body weight gain was 20% below controls over weeks 1–52 in this group, but only slightly lower than controls over the entire 2 year study (not statistically significant). There were no effects on food consumption; the reduced body weight gain was associated with lower mean food efficiency over the first year (although food efficiency over the entire 2 year study was comparable to controls).

### 3.3 Clinical pathology

**Fig. 3:**
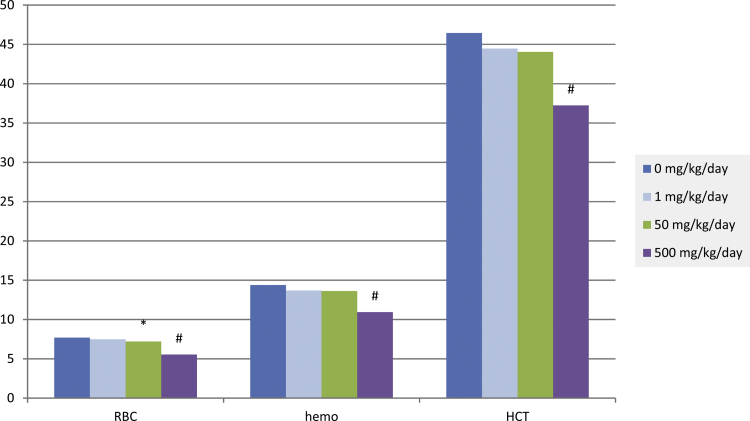
Mean red cell mass parameters for female rats treated with either 0 (control), 1, 50 or 500 mg/kg ammonium 2,3,3,3-tetrafluoro-2-(heptafluoropropoxy)-propanoate for 12 months. RBC = erythrocytes (10^6^/μL); hemo = hemoglobin (g/dL); HCT = hematocrit (%). * = Significantly different from control (*p* < 0.05); # = significantly different from control (*p* < 0.01), *N* = 10.

At the 3, 6, and 12 month intervals, the test substance caused mild but adverse decreases in red cell mass (erythrocytes, hemoglobin, and hematocrit; up to 28% below controls) in females receiving 500 mg/kg (Fig. 3). These changes were associated with an appropriate increase in reticulocytes (up to 106% above controls). There were no effects on erythrocyte morphology.

Statistically significant decreases in erythrocytes, hemoglobin, and hematocrit were also present in males receiving 50 mg/kg at the 3 and 6 month intervals. However, the decreases were small, transient (no statistically significant difference at 12 months), and did not induce a statistically significant increase in reticulocytes.

**Fig. 4:**
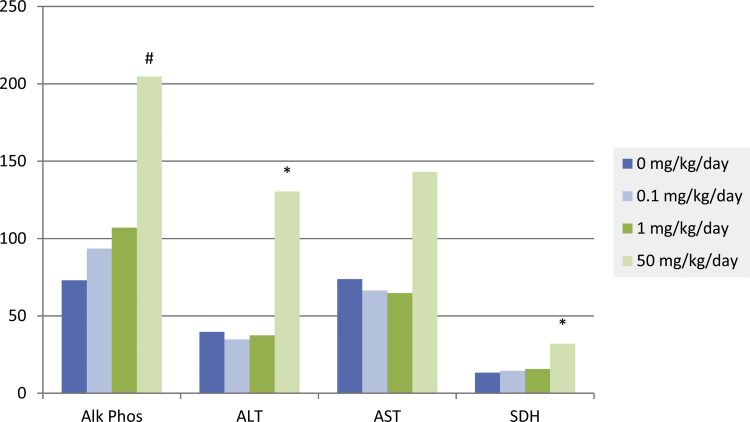
Selected mean clinical chemistry parameters for male rats treated with either 0 (control), 0.1, 1 or 50 mg/kg ammonium 2,3,3,3-tetrafluoro-2-(heptafluoropropoxy)-propanoate for 12 months. Alk Phos = alkaline phosphatase (U/L); ALT = alanine aminotransferase (U/L); AST = aspartate aminotransferase (U/L); SDH = sorbitol dehydrogenase (U/L). * = Significantly different from control (*p* < 0.05); # = significantly different from control (*p* < 0.01), *N* = 10.

Increases in enzymes indicative of liver injury, including mild increases in alkaline phosphatase, alanine aminotransferase, aspartate aminotransferase, and sorbitol dehydrogenase (aspartate aminotransferase was not statistically significant) were observed at 12 months in males at 50 mg/kg (Fig. 4) and were correlated with microscopic findings of minimal cystic degeneration and minimal to mild focal necrosis in the liver. Minimal increases in alkaline phosphatase, unassociated with increases in other liver injury-specific enzymes, were also present in males at this dose level at 3 and 6 months. There were no test article-related liver enzyme changes in males receiving either 0.1 or 1 mg/kg or in females at any dose.

Test substance-associated increases in albumin (up to 16% above controls), decreases in globulin (up to 17% below controls), and increases in the albumin/globulin ratio were present in males at 50 mg/kg and females at 500 mg/kg at various intervals with variable statistical significance. Albumin was also statistically significantly increased in males receiving 1 mg/kg at 12 months (8% above controls) and globulin was statistically significantly decreased transiently (only at 6 months, 6% below controls) in females receiving 50 mg/kg. Minimal, statistically-significant increases in the albumin/globulin ratio were also present in the 1 mg/kg males and the 50 mg/kg females at various intervals.

A minimal diuresis was present at 6 and 12 months in females that received 500 mg/kg, as evidenced by statistically significant increases in urine volume and decreases in urine specific gravity. There were no changes in kidney-related chemistry parameters. This diuresis may be correlated to an increase in the incidence and severity of chronic progressive nephropathy (CPN) observed in this group at the 1-year sacrifice (Table 1).

**Table 1:** Mean liver weight parameters for male rats treated with either 0, 0.1, 1 or 50 mg/kg ammonium 2,3,3,3-tetrafluoro-2-(heptafluoropropoxy)-propanoate and female rats treated with either 0, 1, 50 or 500 mg/kg ammonium 2,3,3,3-tetrafluoro-2-(heptafluoropropoxy)-propanoate for 12 (interim sacrifice) and 24 (terminal sacrifice) months.

Mean liver weights at interim and terminal sacrifice in male and female rats								
Males								
	Interim sacrifice				Terminal sacrifice			
Dose level (mg/kg)	0	0.1	1	50	0	0.1	1	50
*N*	10	10	10	10	15	20	18	18
Absolute (g)	26.9 ± 4.9*	26.1 ± 4.3	25.5 ± 1.9	29.7 ± 5.0	24.3 ± 3.6	26.6 ± 6.4	24.1 ± 5.6	27.1 ± 4.8
%		−3%	−5%	10%		9%	−1%	12%
Liver/body weight (%)	3.2 ± 0.3	3.3 ± 0.3	3.3 ± 0.2	3.7 ± 0.4#	2.9 ± 0.4	3.3 ± 1.3	3.2 ± 0.7	3.2 ± 0.5
%		3%	3%	16%		14%	10%	10%

Females								
	Interim sacrifice				Terminal sacrifice			
Dose level (mg/kg)	0	1	50	500	0	1	50	500
*N*	10	10	10	10	16	22	15	18
Absolute (g)	13.2 ± 2.5	12.0 ± 1.6	14.8 ± 3.6	17.6 ± 2.6#	16.4 ± 3.8	15.8 ± 4.3	17.2 ± 3.0	23.2 ± 8.2#
%		−9%	12%	33%		−4%	5%	41%
Liver/body weight (%)	2.9 ± 0.3	3.0 ± 0.3	3.3 ± 0.4	4.9 ± 0.6#	3.5 ± 0.4	3.3 ± 0.7	3.3 ± 0.6	5.0 ± 0.9#
%		3%	14%	69%		−6%	−6%	43%

# Significantly different from control (*p* < 0.01). %Indicates difference from controls.

* Mean ± standard deviation.

There were no other test article related clinical pathology effects. All other mean and individual clinical pathology parameters were considered within an acceptable range for biological and procedure-related variation.

### 3.4 Organ weights and anatomic pathology observations

No test-substance associated anatomic pathology findings occurred in male rats administered either 0.1 or 1 mg/kg or in female rats administered either 1 or 50 mg/kg. Test substance-associated findings observed in males at 50 mg/kg and in females at 500 mg/kg are described in Section 3.4.1, 3.4.2 and 3.4.3. All other organ weight, macroscopic and non-neoplastic and neoplastic microscopic observations were of the type typically seen in rats of this strain and age, and were considered incidental and not related to compound administration.

#### 3.4.1 Organ weights and macroscopic findings

At the interim necropsy, test substance-associated organ weight effects were limited to increased liver weights (Table 1) in the 50 mg/kg male group (16% above controls relative to body weight) and in the 500 mg/kg female group (69% above controls relative to body weight). In females, liver weight changes correlated with centrilobular hypertrophy microscopically. In one 500 mg/kg female, the macroscopic finding of irregular surface of the kidney correlated with CPN in this animal and was likely test substance-related.

At the terminal sacrifice, no test compound-associated organ weight or macroscopic effects were observed in males. In females, test substance-associated organ weight changes were limited to increased liver weights (Table 1) at 500 mg/kg (43% above controls relative to body weight). Macroscopic findings in this group included tan focus/foci in the liver (8/70 versus 1/70 in controls), mass/nodule in the liver (14/70 versus 0/70 in controls) and irregular surface of the kidney (16/70 versus 0/70 in controls). The organ weight and macroscopic observations in females were correlated to non-neoplastic and neoplastic histological observations at this dose.

#### 3.4.2 Non-neoplastic histological findings

At the interim necropsy, non-neoplastic test substance-associated effects were present in the liver of males at 50 mg/kg and in the liver and kidneys of females at 500 mg/kg. In the liver of males administered 50 mg/kg, histological lesions included minimal focal cystic degeneration (3/10 versus 0/10 in controls) and minimal to mild focal necrosis (5/10 versus 1/10 in controls). In females, histological changes in the liver were limited to minimal to mild hepatocellular hypertrophy in all 10 females receiving 500 mg/kg. A slightly increased incidence and severity of CPN was also observed in interim sacrifice females at 500 mg/kg (Table 2).

**Table 2:** Incidence of chronic progressive nephropathy in the kidneys of female rats treated with either 0 (control), 1, 50 or 500 mg/kg of ammonium 2,3,3,3-tetrafluoro-2-(heptafluoropropoxy)-propanoate for 12 months, *N* = 10.

Incidences and severity of chronic progressive nephropathy in the kidneys of female rats: interim sacrifice				
Dose level (mg/kg)	0	1	50	500

Nephropathy, chronic progressive	6	4	6	9
-Minimal	5	3	4	3
-Mild	0	1	2	6
-Moderate	1	0	0	0

At the terminal sacrifice, non-neoplastic test substance-associated effects were observed histologically at the highest dose level in the liver of both sexes (Table 3) and, additionally, in the kidney (Tables 4), glandular stomach (limiting ridge) and tongue of females at the highest dose (Table 5).

**Table 3:** Test substance-associated non-neoplastic histological lesions in the liver of male rats treated with either 0, 0.1, 1 or 50 mg/kg ammonium 2,3,3,3-tetrafluoro-2-(heptafluoropropoxy)-propanoate and female rats treated with either 0, 1, 50 or 500 mg/kg ammonium 2,3,3,3-tetrafluoro-2-(heptafluoropropoxy)-propanoate for 24 months, *N* = 70.

Summary of test substance-associated non-neoplastic findings in the liver of male and female rats: terminal sacrifice				
Males				
Dose level (mg/kg)	0	0.1	1	50

Degeneration, cystic, focal	24	24	19	42*
Hypertrophy, hepatocyte, centrilobular	0	0	0	7*
Necrosis, hepatocytes, centrilobular	1	0	1	5*

Females				
Dose level (mg/kg)	0	1	50	500

Degeneration, cystic, focal	2	2	2	14*
Hypertrophy, hepatocyte, centrilobular	0	0	3	65*
Hypertrophy, hepatocyte, panlobular	0	0	0	3*
Necrosis, hepatocytes, centrilobular	1	1	4	7*
Necrosis, individual hepatocyte	0	0	0	3*

* Significantly different from control (*p* < 0.05).

**Table 4:** Test substance-associated non-neoplastic histological lesions in the kidney of female rats treated with either 0, 1, 50 or 500 mg/kg ammonium 2,3,3,3-tetrafluoro-2-(heptafluoropropoxy)-propanoate for 24 months, *N* = 70.

Summary of test substance-associated non-neoplastic findings in the kidneys of female rats: terminal sacrifice				
Dose level (mg/kg)	0	1	50	500

Dilation, tubular	4	2	5	28*
Edema, papilla	4	1	2	43*
Hyperplasia, transitional cell	6	3	12	33*
Mineralization, tubular	25	32	28	42*
Necrosis, papillary	0	0	0	16*
Nephropathy, chronic progressive	39	40	41	64*

* Significantly different from control (*p* < 0.05).

**Table 5:** Test substance-associated non-neoplastic histological lesions in the nonglandular stomach and tongue of female rats treated with either 0, 1, 50 or 500 mg/kg ammonium 2,3,3,3-tetrafluoro-2-(heptafluoropropoxy)-propanoate for 24 months, *N* = 70.

Summary of test substance-associated non-neoplastic findings in the nonglandular stomach and tongue of female rats: terminal sacrifice				
Dose level (mg/kg)	0	1	50	500

Stomach, nonglandular				
Hyperplasia, epithelial, limiting ridge	0	0	0	9*

Tongue				
Hyperplasia, squamous cell	2	8	4	13*
Inflammation, subacute/chronic	3	8	4	13*

* Significantly different from control (*p* < 0.05).

In 50 mg/kg males, histopathological effects were limited to the liver and included statistically significant increases in the incidences of focal cystic degeneration, centrilobular hepatocellular hypertrophy, and centrilobular hepatocellular necrosis. These changes were also present in 500 mg/kg females, in addition to low incidences of panlobular hepatocellular hypertrophy and individual cell hepatocellular necrosis (Table 3). Cystic degeneration was characterized by the presence of multilocular cystic spaces containing finely granular or flocculent material without endothelial or epithelial cells lining the spaces. Centrilobular hypertrophy, morphologically consistent with peroxisome proliferation, was characterized by hepatocytes with red granular cytoplasm sometimes containing small amounts of pigment compatible with lipofuscin. Centrilobular hepatocellular necrosis was typically of the coagulative type with strongly eosinophilic-staining cytoplasm and pyknotic nuclei. Panlobular hepatocellular hypertrophy was characterized by enlargement of hepatocytes throughout the entire liver. Individual cell necrosis was characterized by the presence of scattered single hepatocytes with features characteristic of apoptosis.

Kidney changes in females at 500 mg/kg included tubular dilation, edema of the renal papilla, transitional cell hyperplasia in the renal pelvis, tubular mineralization, renal papillary necrosis and CPN. Tubular dilation frequently occurred in an ascending pattern extending from the papilla to the outer cortex, while at other times it was present only in the papilla. Edema of the papilla was characterized by increased rarefaction or myxomatous change in the papillary interstitium, sometimes with polypoid protrusions from the lateral surface of the papilla. The edema and tubular dilation were often associated with hyperplasia of the transitional cell epithelium lining the papilla and pelvis. Small foci of tubular mineralization were often present and, in some animals, necrosis of the tip of the papilla was present.

In addition, in female rats given 500 mg/kg, statistically significant increases in hyperplasia of squamous epithelium were observed in the nonglandular stomach (limiting ridge only) and tongue (in association with subacute/chronic inflammation in the tongue).

A statistically significant increase (42/70 or 61%) in alveolar histiocytosis was present in females at 500 mg/kg and was at the upper end of the historical control range of 9.2–61.7% [22]. The increased incidence of this common background lesion may be secondary to aspiration of dosing formulation at this high concentration; however, a definitive mechanism for this increase could not be determined.

#### 3.4.3 Neoplastic histological findings

Neoplastic test substance-associated effects were observed histologically at the highest dose level in the liver of females and, equivocally, in the pancreas and testes of males at the highest dose (Table 6).

**Table 6:** Test substance-associated neoplastic histological lesions in the liver, pancreas and testes of male rats treated with either 0, 0.1, 1 or 50 mg/kg ammonium 2,3,3,3-tetrafluoro-2-(heptafluoropropoxy)-propanoate and the liver and pancreas of female rats treated with either 0, 1, 50 or 500 mg/kg ammonium 2,3,3,3-tetrafluoro-2-(heptafluoropropoxy)-propanoate for 24 months, *N* = 70.

Summary of test substance-associated neoplastic findings in male and female rats: terminal sacrifice				
Males				
Dose level (mg/kg)	0	0.1	1	50

Liver				
Hepatocellular adenoma	1	2	1	1
Hepatocellular carcinoma	1	0	0	2

Pancreas				
Acinar cell adenoma	0	1	0	3
Acinar cell carcinoma	0	0	0	2
Combined adenoma/carcinoma	0	1	0	5*

Testes				
Leydig cell tumor	4	4	1	8

Females				
Dose level (mg/kg)	0	1	50	500

Liver				
Hepatocellular adenoma	0	0	0	11*
Hepatocellular carcinoma	0	0	0	4*

Pancreas				
Acinar cell adenoma	0	0	0	0
Acinar cell carcinoma	0	0	0	0
Combined adenoma/carcinoma	0	0	0	0

* Significantly different from control (*p* < 0.05).

Compound-related neoplastic changes occurred in the livers of females administered 500 mg/kg and included increased incidences of hepatocellular adenoma and carcinoma (Table 6). These tumors occurred in association with the degenerative and necrotic liver lesions observed at this dose as described above. Hepatocellular tumors and test substance-associated degenerative and necrotic lesions were not observed in females at lower doses and the incidences of hepatocellular tumors were similar in all male groups. In males administered 50 mg/kg, a statistically significant increase in the combined incidence of pancreatic acinar cell adenomas and carcinomas was seen (Table 6), but neither the incidence of adenoma or carcinoma alone was statistically increased, although the incidence of carcinomas (2.9%) was slightly outside the historical range of 0–1.7% [21]. In addition, the incidence of acinar cell hyperplasia was not significantly different from controls in any treated male groups. However, based on the known PPARα agonist activity of the test compound, the marginal increase in pancreatic acinar cell tumors at this dose provides equivocal evidence of a test substance-associated effect. Incidences of proliferative acinar cell lesions in males receiving 0.1 and 1 mg/kg were similar to controls and no acinar cell adenomas or carcinomas were present in females at any dose.

The incidence of Leydig cell adenomas (11.4%) was increased above historical control ranges for this tumor (0–8.3%) [21] in males administered 50 mg/kg, although this increase was not statically significant compared to controls. In addition, a Leydig cell adenoma was present in 1 male at the interim necropsy in the 50 mg/kg group. The incidence of Leydig cell hyperplasia was also increased above historical control range in this group at terminal sacrifice (also 0–8.3%; [22]; although again, this incidence was not statistically significant versus controls. However, comparison to within-study controls was complicated by the fact that controls had a relatively high incidence of Leydig cell hyperplasia (10%). Based on the above considerations and the known activity of PPARα agonists to produce Leydig cell hyperplasia and adenomas in rats, the relationship to the test compound for these lesions was considered equivocal in this study.

All other findings were considered to represent the spontaneous occurrence of neoplasms commonly seen in rats of this strain and age.

## 4 Discussion

Doses employed in this study were designed to and did meet the expectations stated below based on information gathered from previous shorter-term repeated dose studies. The high dose (50 mg/kg in males, 500 mg/kg in females) did produce adverse effects on a number of parameters without producing excessive toxicity which might affect survival. Seven females in the 500 mg/kg group died from papillary necrosis of the kidney but overall survival in this group was similar to that of the other test groups and the controls. The intermediate dose (1 mg/kg in males, 50 mg/kg in females) was selected to either produce slight to minimal effects on target tissues and organs (liver and kidney) and/or to be a no-observed adverse effect level (NOAEL), which it was found to be. The low dose (0.1 mg/kg in males, 1 mg/kg in females) was projected and also found to be a NOAEL in both sexes.

No unusual clinical signs were observed in any of the test groups during the course of the study. Body weights among females in the 500 mg/kg group were significantly reduced, being up to 20% lower than those of the controls. No body weight differences were seen among the male test groups. Food consumption in all test groups was unchanged; however, because of the weight gain depression, females in the 500 mg/kg group showed lower food efficiencies.

Females receiving 500 mg/kg had mild but adverse decreases in red cell mass parameters at all time points tested. These changes were associated with an appropriate increase in reticulocytes and were not accompanied by changes in erythrocyte morphology. Similar decreases in red cell mass parameters were also present in males receiving 50 mg/kg at the 3 and 6 month intervals. However, in males, the decreases were small, transient (there was no statistically significant difference at 12 months), did not induce a statically significant increase in reticulocytes, and values in individual animals were similar to controls. Thus, the changes observed in 50 mg/kg males were considered to be related to treatment but were not considered adverse. This collection of findings suggests red cell loss or hemolysis, although the exact mechanisms involved are unknown. Peroxisome proliferators have been shown to affect iron metabolism, and this mechanism has been hypothesized to be the possible cause of the mild anemia sometimes seen in humans and rats that receive these compounds [14], [15]. No changes in leukocyte numbers or differential counts were seen and coagulation parameters were normal in all test groups.

Clinical chemistry evaluations among males receiving 50 mg/kg revealed mild but adverse increases in enzymes indicative of liver injury, including alkaline phosphatase, alanine aminotransferase, aspartate aminotransferase, and sorbitol dehydrogenase. These enzyme changes correlated with microscopic findings in the liver which consisted of minimal cystic degeneration and minimal to mild focal necrosis. Transient, minimal increases in alkaline phosphatase, unassociated with increases in other liver injury-specific enzymes, were also present in males at this dose level at 3 and 6 months; however, this increase was likely due to hepatic enzyme induction as the test substance has been demonstrated previously to increase total P450 enzyme activity in male rats at 30 mg/kg [12]. These liver enzyme changes were not seen in the male groups receiving either 0.1 or 1 mg/kg or in any of the female test groups.

In addition, a small increase in serum albumin and a decrease in serum globulin were seen in males in the 50 mg/kg group and females in the 500 mg/kg group which led to increases in the albumin/globulin ratio. Slight changes in these serum protein components were also seen in males given 1 mg/kg and females given 50 mg/kg. The test chemical is a peroxisome proliferator and the pattern of change in serum proteins is a well-established response to PPAR activation [8]. Peroxisome proliferators are anti-inflammatory, producing decreases in acute phase proteins (which contribute to the globulin fraction) and increases in the negative acute phase protein, albumin. No adverse biological outcomes have been associated with such changes in these serum proteins. Therefore, the statistically significant changes in albumin/globulin ratio in these groups were considered to be test article-related but nonadverse based on the minimal nature of the changes and lack of association with known adverse outcomes.

The minimal diuresis observed in females that received 500 mg/kg at 6 and 12 months was not associated with changes in kidney-related chemistry parameters. This diuresis may be correlated to an increase in the incidence and severity of CPN observed in this group at the 1-year sacrifice (Table 1).

Liver weights were increased in high dose male and female rats at the interim sacrifice and in high dose females only at terminal sacrifice. Microscopic evaluation of the liver revealed changes only in the high dose males and females. These changes occurred at both the terminal sacrifice and, in a less progressed form, at the interim sacrifice and consisted of microscopic observations including focal cystic degeneration, centrilobular (and panlobular in females only) hepatocellular hypertrophy, centrilobular hepatocellular necrosis, and individual cell necrosis (females only). In addition, the incidences of both adenoma and carcinoma in the liver exceeded the historical control range in females administered 500 mg/kg (only a few hepatocellular tumors occurred in males with the incidence being essentially the same between the controls and the test groups) and the increased incidences of hepatocellular tumors in females occurred in association with degenerative/necrotic liver changes. This battery of liver changes is consistent with PPARα activation.

Other non-neoplastic microscopic observations included findings in the kidneys of females at 500 mg/kg at both the interim (in a less severe form) and terminal sacrifices, and included tubular dilation, edema of the renal papilla, transitional cell hyperplasia in the renal pelvis, tubular mineralization, renal papillary necrosis, and CPN. In addition, the nonglandular stomach (limiting ridge only) and the tongue had statistically significantly increased incidences of hyperplasia of squamous epithelium in females at 500 mg/kg at the terminal sacrifice only. The lesion in the tongue was associated with subacute/chronic inflammation.

In males administered 50 mg/kg, the incidences of pancreatic acinar cell adenoma/carcinoma combined, but not adenoma or carcinoma alone, were statistically significantly increased and the incidence of carcinoma was slightly outside the historical control range. Since pancreatic acinar cell hyperplasia and adenoma in rats occur along a continuum, the incidence of acinar cell hyperplasia would have been expected to be increased if a test-article related increase in acinar cell adenoma was found. However, the incidences of acinar cell hyperplasia were not significantly different from controls in any of the treated male groups. Based on these considerations, and the known PPARα agonist activity of the test article, the marginal increase in pancreatic acinar cell tumors in this group provides equivocal evidence of a test article-related effect.

The incidences of Leydig cell adenoma of the testes were increased above historical control range in the 50 mg/kg group at terminal sacrifice, although this finding was not statistically significant. In addition, a single Leydig cell adenoma was also present in 1 male from this group at the interim sacrifice. Incidences of Leydig cell hyperplasia were also elevated in this group above historical control range, although this, too, was not statistically significant compared to controls. Since PPARα agonists are known to produce proliferative Leydig cell lesions (hyperplasia and adenoma) in the testes of rats, a relationship to treatment for these findings in the 50 mg/kg group cannot be ruled out.

The test chemical belongs to a class of compounds known as peroxisome proliferators (PPARα agonists) which are known to produce liver, pancreatic, and testicular tumors in rats and liver tumors in mice [3], [16]. However, these compounds have not been shown to be carcinogenic in other species including humans [16], [7]. Based on the extensive research into the comparative biology of peroxisome proliferator-induced hepatic carcinogenesis, the induction of liver tumors in rodents by non-genotoxic peroxisome proliferators (this compound was shown to be inactive in a battery of genotoxicity assays) is not considered relevant to humans [23], [7], [17]. While there is less definitive mechanistic data on the role PPARα plays in the induction of pancreatic acinar cell tumors in rats, the available data involving altered bile flow and increased cholecystokinin suggests that this mode of action is also likely to be non-relevant for humans [16]. While less robust, research considering comparative biology and mechanism of action of Leydig cell tumor induction in rodents by a wide variety of chemical classes suggests these tumors most likely have low relevance to humans [25], [6].

The findings of this study with this fluorochemical polymerization processing aid can be compared to the results from similar studies with other fluorochemical polymerization processing aids (and PPARα agonists). The perfluorinated C6 chain chemical, perfluorohexanoic acid (PFHxA), showed no evidence of an adverse effect in male rats given oral doses of up to 100 mg/kg for 2 years [18]. Females given either 5, 30, or 200 mg/kg showed a dose-related decrease in survival and, at 200 mg/kg, had kidney lesions characterized by papillary necrosis and tubular degeneration. No evidence for a tumorigenic response was found. For a sulfonated fluorochemical, perfluorooctane sulfonate (PFOS), when fed in the diet of rats for 2 years at doses equivalent to approximately 0.02, 0.1, 0.2, and 1 mg/kg, the primary organ affected was the liver with changes consisting of hepatocellular centrilobular hypertrophy, eosinophilic granules, and vacuolation [27]. Neoplastic lesions consisted of an increase of hepatocellular adenomas in male rats; females showed no increase in the incidence of neoplasia. With the ammonium salt of perfluorooctanoic acid (APFO), when fed for 2 years to male and female rats at doses approximately equivalent to 1.5 and 15 mg/kg, the liver was the target organ with an increase in the incidence of hepatocellular hypertrophy, monocellular infiltration, and hepatocellular vacuolation [5]. The neoplastic changes in this study were restricted to Leydig cell adenomas of the testes. In a follow-up mechanistic study, male rats treated at 300 ppm of APFO for 2 years (equivalent to a daily dose of 15 mg/kg) showed increased tumor incidences in the liver (hepatocellular adenomas), pancreas (acinar cell adenomas), and testes (Leydig cell adenomas) [4].

The neoplastic changes found with this fluorochemical are consistent with many of those seen with the compounds above and with other peroxisome proliferating chemicals, such as clofibrate [26], HCFC-123 [19], gemfibrozil, and diethyl-hexyl phthalate [6], when tested in rodents. Although hepatocellular adenomas were not identified in males in this study, the neoplastic pattern is similar to that seen in rats administered APFO but was seen at a dose somewhat higher (50 versus 15 mg/kg). In female rats, no increase in liver neoplasia was reported with APFO while this chemical produced an increase in both adenomas and carcinomas, but the response was seen only when the dose was considerably higher (500 versus 15 mg/kg) than the dose used in the APFO study.
